# Expectancy of asthma phenotype having different polymorphism of PSMA6, PSMC6, PSMA3 proteasomal genes: Lithuanian study

**DOI:** 10.1186/1939-4551-8-S1-A154

**Published:** 2015-04-08

**Authors:** Laura Tamasauskiene, Zivile Zemeckiene, Raimundas Sakalauskas, Brigita Sitkauskiene

**Affiliations:** 1 Lithuanian University of Health Sciences, Lithuania

## Background

According to the scientific literature, proteasomes and proteasomal genes which encode them may be important in development of different chronic inflammatory diseases, including asthma, through the activation of the NF-κB protein. We aimed to investigate expectancy of asthma in subjects with different polymorphism of proteasomal genes PSMA6 (rs1048990 and rs2277460), PSMC6 (rs2295827 and rs2295827) and PSMA3 (rs2348071) and to analyze possible relation to gender, allergic status and lung function.

## Methods

296 individuals were involved in this study: 146 with mild to moderate asthma (according to GINA) and 150 healthy subjects. Allergic status was evaluated using skin prick test, blood eosinophil count and immunoglobulin E (IgE) in serum. Lung function was measured by standard spirometry. Alleles and genotypes of PSMA6 (rs1048990 and rs2277460), PSMC6 (rs2295827 and rs2295827) and PSMA3 (rs2348071) were evaluated using allele specific amplification and cleaved amplified polymorphic sequence methods. DNA from peripheral blood was extracted using Qiagen (Germany) DNA blood mini kit (according to standardised protocol).

## Results

Expectancy of asthma was similar in studied subjects despite their proteasomal genes polymorphism: PSMA6 rs2277460 (CC) B=1.53, PSMA6 rs1048990 (CC) B=0.47, PSMA6 rs1048990 (CG) B=0.52, PSMC6 rs2295826 (AA) B=1.98, PSMC6 rs2295826 (AG) B=1.66, PSMC6 rs2295827 (CC) B=2.08, PSMC6 rs2295827 (CT) B=1.74, PSMA3 rs2348071 (GG) B=1.20 and PSMA3 rs2348071 (GA) B=1.43. Asthma was diagnosed at the same frequency in individuals having common genotype or at least one rare genotype (rs1048990 GG, rs2295826 GG, rs2295827 TT and rs2348071 AA) (50.4% vs. 41.7%). However, rs1048990 CG genotype was more common in males with asthma than in healthy males (37.3% vs. 17.0%, p<0.05). Analysis of blood eosinophil count showed significantly higher number of these cells in asthmatics with common genotype than in asthmatics with rare genotype (5.4% vs. 3.0%, p<0.05) and positive relation to this type of proteasomal genes polymorphism (r=0.2, p<0.05). Positive skin prick test, total IgE level and lung function results did not differ significantly in these groups.

## Conclusions

According to our study results, asthma is expected at the same frequency in Lithuanian subjects despite their proteasomal genes polymorphism type; however rs1048990 CG genotype is more prevalent in males with asthma. Common genotype polymorphism is related to eosinophilic asthma phenotype.

